# Commentary: Case Report: Multimodal management of duodenal metastasis from hepatocellular carcinoma complicated by intractable hemorrhage

**DOI:** 10.3389/fimmu.2026.1914627

**Published:** 2026-07-27

**Authors:** Xiaoli Huang, Tao Xu, Min Du

**Affiliations:** Department of Oncology, The General Hospital of Western Theater Command, Chengdu, Sichuan, China

**Keywords:** duodenal metastasis, gastrointestinal hemorrhage, hepatocellular carcinoma, immunotherapy, transarterial embolization

## 1 Introduction

Hepatocellular carcinoma (HCC) remains one of the leading causes of cancer-related mortality worldwide, particularly in regions with a high prevalence of chronic hepatitis B virus infection (1, 2). Recent advances in antiviral therapy, hepatic resection, locoregional interventions, and immune-based systemic therapies have substantially improved survival outcomes for patients with advanced-stage disease (3). As patient survival continues to improve, uncommon metastatic patterns and late-stage complications that were previously encountered only sporadically may become increasingly recognized in clinical practice, although robust epidemiological evidence supporting an increasing incidence remains limited (4). These evolving clinical patterns present new diagnostic and therapeutic challenges that are not fully addressed by current HCC management strategies or surveillance recommendations, highlighting the need to reconsider how atypical metastatic manifestations should be recognized and managed in long-term survivors.

In a recently published case report, Zhang and colleagues (4) described a patient with HCC complicated by portal vein tumor thrombus (PVTT) who developed duodenal metastasis associated with recurrent, life-threatening gastrointestinal bleeding. Following pathological confirmation by endoscopic biopsy, the patient was successfully managed with transarterial embolization for hemorrhage control, followed by immunotherapy, ultimately achieving durable disease control and significant tumor regression. Beyond its clinical rarity, this case provides valuable insights into the diagnosis and management of gastrointestinal metastatic disease in patients with advanced HCC. More importantly, it raises broader questions regarding the early recognition of rare metastatic sites, the optimal integration of diagnostic modalities, the coordination of multidisciplinary care, and the evolving role of locoregional and systemic therapies in complex clinical scenarios. Importantly, as a single-patient experience, its principal value lies in generating clinically relevant hypotheses and informing multidisciplinary clinical decision-making rather than establishing a definitive therapeutic strategy. Accordingly, this commentary aims not only to discuss the clinical lessons offered by this case but also to examine its broader implications for contemporary HCC management and to identify areas requiring further clinical investigation.

## 2 Why is duodenal metastasis often overlooked?

The gastrointestinal tract is an uncommon site of HCC dissemination (5). While the lungs, bones, and lymph nodes account for the majority of extrahepatic metastases, gastrointestinal involvement occurs in less than 1% of cases (6). Duodenal metastasis is even rarer, resulting in limited clinical awareness and a low index of suspicion among treating physicians. Consequently, gastrointestinal involvement is seldom considered during routine surveillance unless patients develop overt gastrointestinal symptoms, which may contribute to delayed recognition in clinical practice.

From a hepatobiliary surgical perspective, duodenal involvement is unlikely to represent a random event. Several mechanisms have been proposed, including direct tumor invasion, hematogenous dissemination, and lymphatic spread (6, 7). Tumors located in the posterior right hepatic segments or caudate lobe may be particularly prone to local extension because of their close anatomical relationship with the duodenum. In addition, the presence of PVTT reflects aggressive tumor biology characterized by vascular invasion and an increased propensity for metastatic dissemination. Although these clinicopathological features may help identify patients at increased risk, they have not been incorporated into current surveillance strategies, and no consensus exists regarding whether selected high-risk patients should undergo additional evaluation for gastrointestinal involvement. Current international HCC guidelines primarily emphasize surveillance for intrahepatic recurrence and common sites of extrahepatic disease, whereas specific recommendations regarding surveillance for gastrointestinal metastases are lacking.

The patient described by Zhang et al. exhibited multiple risk factors for advanced disease progression, including extensive PVTT, repeated locoregional interventions, and persistent tumor progression (4). Nevertheless, the diagnosis of duodenal metastasis was delayed for more than two years. This observation highlights a critical challenge in contemporary HCC management: as survival improves, clinicians must broaden their understanding of disease evolution and remain vigilant for atypical metastatic presentations that may be encountered during long-term follow-up. Although it is biologically plausible that prolonged survival may increase opportunities to recognize such uncommon metastatic manifestations, current epidemiological evidence remains insufficient to support a definitive temporal trend. More importantly, this case raises an unresolved clinical question: whether prolonged survival achieved through modern multimodal therapies should prompt a more individualized surveillance strategy for patients with high-risk features, rather than relying solely on conventional follow-up protocols. Addressing this question will require prospective studies to define which patients are most likely to benefit from intensified surveillance while avoiding unnecessary investigations in those at low risk.

## 3 What should clinicians consider in recurrent gastrointestinal bleeding?

One of the most striking aspects of this case is the prolonged diagnostic delay despite repeated episodes of gastrointestinal hemorrhage. In patients with cirrhosis and portal hypertension, hematemesis and melena are commonly attributed to esophagogastric variceal bleeding, portal hypertensive gastropathy, or peptic ulcer disease. Consequently, the diagnostic evaluation of gastrointestinal bleeding in patients with HCC is often focused primarily on portal hypertension-related complications, potentially obscuring alternative etiologies. This diagnostic tendency may be particularly problematic in patients with advanced disease, in whom multiple causes of gastrointestinal bleeding may coexist and should therefore be evaluated systematically rather than sequentially.

Notably, early endoscopic examinations in this patient had already demonstrated subtle mucosal abnormalities. However, the absence of distinctive endoscopic features and the lack of corresponding radiological findings contributed to delayed recognition of metastatic disease. Definitive diagnosis was established only after the lesion enlarged sufficiently to produce active bleeding and mass formation. This sequence of events highlights an important diagnostic challenge: early metastatic lesions may initially present with subtle or nonspecific endoscopic findings that are easily overlooked, particularly when cross-sectional imaging remains unremarkable. Such discordance between clinical symptoms, endoscopic appearances, and radiological findings should prompt continued diagnostic reassessment rather than premature exclusion of malignant involvement.

This clinical course underscores an important principle: gastrointestinal bleeding in patients with advanced HCC should not automatically be attributed to portal hypertension. In patients with PVTT, progressive intrahepatic disease, unexplained recurrent bleeding, or inadequate response to conventional hemostatic measures, gastrointestinal metastasis should be actively considered. When focal mucosal elevations, ulcerative lesions, or mass-like abnormalities are identified endoscopically, histopathological confirmation should be pursued whenever feasible. Tissue diagnosis remains the cornerstone for distinguishing metastatic disease from benign gastrointestinal pathology. Although current evidence is insufficient to support routine endoscopic surveillance for all patients with advanced HCC, this case suggests that repeat endoscopic evaluation may be warranted in carefully selected patients with persistent or recurrent bleeding, unexplained anemia, progressive disease despite standard therapy, or persistent clinical suspicion despite negative imaging findings. Likewise, endoscopic biopsy should be considered whenever suspicious mucosal abnormalities persist or evolve during follow-up, provided that the anticipated diagnostic benefit outweighs the procedural risk. Future studies are needed to establish evidence-based criteria for patient selection and surveillance intervals.

## 4 Endoscopy versus computed tomography: which modality detects early disease more effectively?

Another notable observation from this case is the discrepancy between endoscopic and radiological detection of the metastatic lesion. Contrast-enhanced computed tomography (CT) remains a cornerstone of HCC diagnosis, staging, and treatment assessment. However, its sensitivity may be limited when metastatic lesions are confined to the mucosal or submucosal layers and have not yet produced substantial wall thickening or characteristic enhancement patterns. Similarly, magnetic resonance imaging (MRI), despite its excellent soft-tissue contrast and value in characterizing hepatic lesions, may also have limited sensitivity for detecting early superficial gastrointestinal involvement. Functional imaging modalities such as positron emission tomography/computed tomography (PET/CT) may identify metabolically active metastatic disease in selected patients, although their routine role in detecting gastrointestinal metastases from HCC remains uncertain because of variable fluorodeoxyglucose avidity and limited supporting evidence.

By contrast, endoscopy permits direct visualization of subtle mucosal changes and facilitates immediate tissue sampling. In the present case, abnormal duodenal mucosal findings were detected endoscopically long before corresponding abnormalities became apparent on CT imaging. This finding highlights the unique value of endoscopic surveillance in selected high-risk patients. Endoscopic ultrasound (EUS) may further complement conventional endoscopy by providing detailed assessment of mural invasion and adjacent structures while enabling tissue acquisition in appropriately selected cases. In addition, contrast-enhanced ultrasound (CEUS), although primarily used for characterizing focal hepatic lesions rather than gastrointestinal metastases, may offer complementary information in selected clinical scenarios. Nevertheless, evidence supporting the routine use of these modalities for evaluating suspected gastrointestinal metastases from HCC remains limited.

Importantly, endoscopy and cross-sectional imaging should be viewed as complementary rather than competing diagnostic modalities. While CT provides comprehensive anatomical assessment and evaluation of disease extent, endoscopy offers superior sensitivity for detecting early mucosal lesions and enables immediate histopathological confirmation. MRI, PET/CT, EUS, and CEUS may each provide additional diagnostic information in selected clinical scenarios, although their optimal roles in evaluating suspected gastrointestinal metastases from HCC remain to be established. Therefore, negative imaging findings should not preclude further endoscopic evaluation when clinical suspicion remains high. Rather than relying on any single diagnostic modality, clinicians should integrate clinical presentation, imaging findings, endoscopic assessment, and pathological confirmation to guide individualized diagnostic decision-making. Rather than supporting routine endoscopic surveillance for all patients with advanced HCC, this case suggests that endoscopic evaluation should be considered in carefully selected patients presenting with recurrent gastrointestinal bleeding, unexplained anemia, or persistent clinical suspicion despite negative cross-sectional imaging. Future studies should define not only which patients are most likely to benefit from selective endoscopic evaluation but also how multimodal imaging techniques can be optimally incorporated into individualized diagnostic pathways.

## 5 Is the role of interventional therapy limited to hemorrhage control?

The primary objective of treatment in patients presenting with tumor-related gastrointestinal bleeding is undoubtedly hemostasis. However, the clinical significance of transarterial embolization extends beyond immediate bleeding control. In the reported case, repeated superselective embolization procedures successfully achieved durable hemostasis and eliminated transfusion dependence. More importantly, stabilization of the patient’s clinical condition enabled subsequent systemic antitumor therapy (8). This sequence underscores an important principle in the management of advanced malignancies: effective control of life-threatening complications may be a prerequisite for delivering potentially beneficial oncologic treatment rather than an endpoint of care in itself.

This concept has important implications for the management of advanced HCC. In many patients, the major barrier to effective treatment is not the absence of therapeutic options but rather the inability to tolerate therapy because of severe complications such as hemorrhage, anemia, malnutrition, or infection. Consequently, interventional hemostatic procedures may serve not merely as rescue interventions but as bridging strategies that create opportunities for definitive oncologic treatment. Importantly, successful bridging requires coordinated multidisciplinary care extending beyond interventional radiology. Nutritional optimization, transfusion support, infection control, symptom management, and careful assessment of functional status are often equally important in restoring treatment eligibility. Furthermore, palliative radiotherapy may represent an alternative or complementary hemostatic option in selected patients with unresectable bleeding lesions, particularly when embolization is technically infeasible, contraindicated, or fails to achieve durable bleeding control. The optimal selection among these approaches should be individualized within a multidisciplinary team.

Viewed from this perspective, transarterial embolization functions as an integral component of comprehensive cancer care rather than a purely supportive measure. By restoring clinical stability, it may facilitate the timely initiation or continuation of subsequent systemic therapy in appropriately selected patients. However, in the present case, the favorable clinical outcome should not be attributed to embolization alone, as hepatic arterial infusion chemotherapy (HAIC) and immunotherapy were subsequently administered as part of a multidisciplinary treatment strategy. Accordingly, embolization should be viewed as one component of a broader therapeutic continuum rather than an isolated intervention, with its clinical value depending largely on how effectively it is integrated with subsequent oncologic management.

Accordingly, the principal contribution of transarterial embolization in this setting may lie in creating a therapeutic window for further oncologic management rather than directly determining long-term tumor control. The relative contributions of embolization, HAIC, and immunotherapy cannot be distinguished on the basis of a single case, and this integrated treatment approach should therefore be interpreted as hypothesis-generating rather than evidence of therapeutic superiority. Future prospective studies are needed to clarify how embolization can be optimally incorporated into multidisciplinary treatment pathways, including its timing relative to systemic therapy and its role alongside other supportive and locoregional interventions.

## 6 Can locoregional therapy synergize with immunotherapy?

One notable observation from this case is the substantial antitumor response observed following successful hemorrhage control. After embolization, the patient received tislelizumab in combination with HAIC, resulting in marked regression of the primary hepatic lesion, PVTT, and duodenal metastasis. Although causality cannot be established from a single case, these findings are consistent with growing interest in the potential interactions between locoregional therapies and cancer immunotherapy. Importantly, the favorable clinical outcome observed in this patient should be interpreted as a clinical observation rather than evidence of a causal therapeutic interaction.

Accumulating preclinical data suggest that embolization-based therapies may induce immunogenic tumor cell death, promote tumor antigen release, enhance antigen presentation, and reshape the tumor immune microenvironment. In parallel, treatment-induced hypoxia may increase PD-L1 expression, potentially augmenting the effectiveness of immune checkpoint blockade (9, 10). In addition, the release of tumor-associated antigens following embolization may facilitate dendritic cell activation and cross-presentation to tumor-specific CD8^+^ T cells, while local inflammatory signaling may further promote immune-cell recruitment within the tumor microenvironment. Collectively, these immunological alterations provide a biologically plausible rationale for combining locoregional therapy with immune checkpoint inhibition, although the precise mechanisms remain to be fully elucidated. While these mechanistic findings provide a strong biological foundation, they do not by themselves establish clinical efficacy, and their translational relevance requires confirmation in well-designed prospective studies.

Consequently, combinations such as TACE plus immunotherapy or HAIC plus immunotherapy have emerged as major areas of investigation in advanced HCC. Early clinical studies have reported encouraging therapeutic activity with several locoregional therapy–immunotherapy combinations; however, the relative contribution of each treatment component remains difficult to determine because multimodal regimens are frequently administered concurrently or sequentially. The present case raises the possibility that similar synergistic effects may also be relevant in patients with uncommon metastatic manifestations. Nevertheless, because the clinical observations derive from a single patient treated with embolization, HAIC, and immunotherapy in sequence, the individual contribution of each therapeutic component cannot be determined. Accordingly, the proposed interaction between locoregional therapy and immune checkpoint blockade should be regarded as hypothesis-generating rather than confirmatory and requires validation in prospective mechanistic and clinical studies. Future research should focus not only on confirming whether such synergy exists but also on identifying the patients most likely to benefit, determining the optimal sequencing of locoregional and systemic therapies, and developing predictive biomarkers to guide individualized treatment strategies.

## 7 Broader implications for the management of advanced HCC

The most important message conveyed by this case extends beyond the successful application of any single therapeutic modality. Rather, it illustrates the evolving paradigm of multidisciplinary care in advanced HCC. The patient’s clinical course involved nearly every major component of modern HCC management, including oncologic assessment, PVTT evaluation, endoscopic investigation, pathological confirmation, interventional radiology, and systemic therapy. The favorable outcome was not attributable to any individual intervention but rather to the coordinated integration of multiple specialties and the continuous adaptation of treatment strategies throughout the disease course. Importantly, this case also reflects a broader shift in HCC management, whereby prolonged survival achieved through advances in systemic and locoregional therapies increasingly exposes clinicians to uncommon metastatic patterns and complex treatment-related challenges that require coordinated longitudinal care rather than isolated therapeutic interventions.

As HCC management enters an era of increasingly personalized and multimodal therapy, collaboration among hepatobiliary surgeons, gastroenterologists, interventional radiologists, medical oncologists, pathologists, and imaging specialists has become essential. Depending on individual clinical circumstances, multidisciplinary teams may also benefit from the involvement of radiation oncologists, palliative care specialists, nutrition support teams, and other supportive care professionals, particularly when managing patients with recurrent bleeding, compromised performance status, or advanced metastatic disease. Such multidisciplinary coordination is likely to play a decisive role in improving outcomes for patients with complex and advanced disease. Beyond optimizing treatment delivery, multidisciplinary care may facilitate timely reassessment of therapeutic goals, appropriate sequencing of locoregional and systemic therapies, and individualized supportive care throughout the disease course. At the same time, it should be recognized that the multidisciplinary strategy described in this case reflects the clinical management of a single patient. Although the favorable outcome provides valuable clinical insights, it should primarily be viewed as generating hypotheses for future multidisciplinary research rather than establishing an evidence-based therapeutic framework. Whether current surveillance strategies, multidisciplinary care pathways, and treatment algorithms should be further refined for patients with prolonged survival and atypical metastatic manifestations remains an important question for future investigation.

## 8 Conclusions

As therapeutic advances continue to extend survival in patients with advanced HCC, rare metastatic manifestations and atypical complications may be encountered more frequently in clinical practice, although further epidemiological studies are needed to clarify whether their incidence is truly increasing. Although duodenal metastasis remains uncommon, its clinical consequences can be severe, including recurrent hemorrhage, obstruction, and substantial impairment of quality of life. Heightened awareness of gastrointestinal metastatic disease is therefore warranted, particularly in patients with PVTT, prolonged treatment histories, and unexplained gastrointestinal bleeding. Rather than supporting routine surveillance for all patients with advanced HCC, this case highlights the importance of maintaining a high index of suspicion in carefully selected high-risk patients and adopting an individualized diagnostic approach when conventional evaluations fail to adequately explain the clinical presentation.

Timely endoscopic evaluation, accurate pathological diagnosis, effective interventional hemostasis, and the seamless integration of systemic therapy collectively represent the foundation of successful management in such challenging cases. More broadly, this case underscores the importance of integrating multimodal diagnostic assessment, multidisciplinary decision-making, and individualized supportive care throughout the disease course. Importantly, the principal contribution of the original case is not to establish a definitive therapeutic strategy but to generate clinically relevant hypotheses regarding multidisciplinary management and the potential integration of locoregional therapy with immunotherapy. These observations should therefore be interpreted cautiously and warrant further validation through mechanistic investigations and prospective clinical studies. Future research should focus on refining risk-adapted surveillance strategies, optimizing the integration and sequencing of diagnostic and therapeutic modalities, and identifying patients most likely to benefit from individualized multidisciplinary management. Ultimately, the principal contribution of this case lies not in defining a new standard of care, but in stimulating further investigation into individualized diagnostic and multidisciplinary management strategies for patients with rare gastrointestinal metastatic manifestations of HCC.
